# Diabetes among Patients with Overt Primary Hypothyroidism Visiting the Outpatient Department of General Medicine of a Tertiary Care Centre: A Descriptive Cross-sectional Study

**DOI:** 10.31729/jnma.8161

**Published:** 2023-05-31

**Authors:** Sadikshya Poudel, Mubashir Angolkar, Arif Maldar

**Affiliations:** 1 Department of Public Health, Jawaharlal Nehru Medical College, Karnatak Lingayat Education University, Belagavi, Karnataka, India; 2 Department of General Medicine, Jawaharlal Nehru Medical College, Karnatak Lingayat Education University, Belagavi, Karnataka, India

**Keywords:** *diabetes mellitus*, *hypertension*, *hypothyroidism*, *thyroid disorder*

## Abstract

**Introduction:**

Thyroid diseases are one of the commonest endocrine disorders and hypothyroidism is the commonest among them. There are many publications about hypothyroidism prevalence in diabetes, however, reports of diabetes in hypothyroidism are scarce. This study aimed to find out the prevalence of diabetes among patients with overt primary hypothyroidism visiting the Outpatient Department of General Medicine of a tertiary care centre.

**Methods:**

A descriptive cross-sectional study was conducted among adults with overt primary hypothyroidism who attended the Department of General Medicine of a tertiary care centre. Data from 1 November 2020 to 30 September 2021 were collected between 1 December 2021 and 30 December 2021 from the hospital records. Ethical approval was obtained from Institutional Review Committee (Reference number: MDC/DOME/258). Convenience sampling method was used. Out of all patients with different thyroid disorders, consecutive patients with overt primary hypothyroidism were included. The patients with incomplete information were excluded. Point estimate and 95% Confidence Interval were calculated.

**Results:**

Among total 520 patients with overt primary hypothyroidism, the prevalence of diabetes was 203 (39.04%) (34.83-43.25, 95% Confidence Interval), with 144 (70.94%) in female and 59 (29.06%) in male. Among 203 hypothyroid patients with diabetes, the proportion of female was more than that of male.

**Conclusions:**

The prevalence of diabetes among patients with overt primary hypothyroidism was higher than the other studies done in similar settings.

## INTRODUCTION

Apart from diabetes, thyroid diseases are among the commonest endocrine disorders worldwide and hypothyroidism is one of the commonest thyroid diseases.^1,2^ Hypothyroidism is common in the region. Hypothyroidism prevalence is reported from 8.8 to 12.4% in the region.^1-3^

Though there are many publications about the prevalence of hypothyroidism in people with diabetes,^4,5^ Early diagnosis and intervention can be done to prevent dreadful complications. There are limited studies regarding this topic.

This study aimed to find out the prevalence of diabetes among patients with overt primary hypothyroidism presented to the Outpatient Department of General Medicine of a tertiary care centre.

## METHODS

This was a descriptive cross-sectional study conducted on the patients with overt primary hypothyroidism who attended the Department of General Medicine of KLE's (Karnataka Lingayat Education) Dr Prabhakar Kore Charitable Hospital and Medical Research Centre (MRC) Belagavi, Karnataka, India. Data from 1 November 2020 to 30 September 2021 were collected between 1 December 2021 and 30 December 2021 from the hospital records. The duration of the study was from The ethical approval was obtained from the Institutional Review Committee of JNMC, KAHER, Belagavi (Reference number: M DC/DO ME/258). Due permission was taken from the hospital administration for access to the relevant data. Out of all the patients with different thyroid disorders, the patients with overt primary hypothyroidism were diagnosed with low thyroxine (T4) and elevated thyroid-stimulating hormone (TSH) were included in the study.^6^ The patients with incomplete information were excluded. The sample size was calculated using the formula:


$$\begin{array}{ll} \text{n} & ={\text{Z}}^{2}\times \frac{\text{p}\times \text{q}}{{\text{e}}^{\text{2}}}\\ & =1.96^{2}\times \frac{0.50\times 0.50}{0.05^{2}}\\ & =385 \end{array}$$

Where,

n = minimum required sample size
Z = 1.96 at 95% Confidence Interval (CI)
p = prevalence of diabetes taken as 50% for maximum sample size
q = 1-P
e = margin of error, 5%

The calculated sample size is 385. Adding 20% nonresponse rate, required sample size was 462. However, we took 520 patients. Convenience sampling method was used. The data was extracted from electronic medical record system of the hospital. The data regarding age, gender and diagnoses of diabetes and hypertension were collected and filled up in the proforma for each patient by the investigator.

Data were then entered in Microsoft Excel version 16.0 and analyzed using IBM Statistics SPSS version 20.0. Point estimate and 95% CI were calculated.

## RESULTS

Among total 520 patients with overt primary hypothyroidism, the prevalence of diabetes was 203 (39.04%) (34.83-43.25, 95% CI), with 144 (70.94%) in female and 59 (29.06%) in male. Among 203 hypothyroid patients with diabetes, the proportion of female was more than that of male (Table 1).

**Table 1:** Genderwise distribution of patients with diabetes among overt primary hypothyroid (n= 203).

Gender	n (%)
Female	144 (70.94)
Male	59 (29.06)

The mean age of the hypothyroid patients with diabetes was 59.37±13.124 years, with 57.83±12.438 years of female and 63.12±14.082 years of male.

## DISCUSSION

In our study, the prevalence of diabetes among overt primary hypothyroid was 39.04% with 70.94% in female and 29.06% in male. Other studies showed the prevalence of diabetes in hypothyroidism is 18.7% to 20% in hospital-based studies,^6,7^ and 13.5% in a health clinic-based study in India.^8^ A community-based study in Pakistan reports the prevalence of diabetes in patients with hypothyroidism as 16.5%.^9^ In summary, the prevalence of diabetes in the hypothyroid patients in our study is higher than that reported in similar settings.

A meta-analysis reports that increased TSH and decreased FT3 and FT4 are associated with a higher risk of type 2 diabetes in a J-shaped and inverted J-shaped relationship, respectively.^10^ The increased diabetes prevalence in hypothyroid patients could be due to the three factors such as the three groups of risk-factors, namely ageing, sedentary life, and obesity; maternal malnutrition; and maternal hyperglycemia during pregnancy,^11-13^ or other autoimmune and metabolic factors. Review articles on the relationship between thyroid disease and diabetes and metabolic diseases discuss the other possible metabolic and autoimmune factors involved in such a relationship.^14,15^ The higher prevalence of diabetes among hypothyroid patients in male in our study indicates the possibility of general metabolic factors rather than autoimmune factors. No significant association was found between gestational diabetes mellitus and thyroid disorders and the risk of developing type 2 diabetes.^16^

Patients with hypothyroidism with diabetes are more likely to visit hospitals than hypothyroid patients without diabetes due to the need of frequent monitoring and managing diabetes and its complications. Moreover, the proportion of diabetic patients may differ across hospitals depending on the hospital's practice, population coverage, and community health services. We recommend larger studies of diabetes and thyroid function tests in the general population, of diabetes in patients with primary overt hypothyroidism in the community, and of thyroid function tests in people with diabetes in the community to reach a consensus in the first place whether there is a such high prevalence of diabetes in the patients with overt primary hypothyroidism or not. Whether the such association is caused by the three groups of factors mentioned above,^11-13^ or by other autoimmune and metabolic factors,^14,15^ may also be then subsequently considered.

Our study has some limitations. Since this study is based on a single hospital, so the results may not be generalized to the whole population. Due to the descriptive nature of our study, we could not establish causality. The sample size particularly of the male patients is relatively less. This study also did not examine the duration and treatment of diabetes, the patients with incomplete information, and other risk factors like family history, smoking, body mass index, physical activity, dietary habits, and autoimmune factors. As our study is hospital-based one, the patients of hypothyroidism with diabetes may be more likely to come to the hospital than the patients without diabetes and that may have increased the proportion of diabetes in our hospital-based patients.

## CONCLUSIONS

The prevalence of diabetes among patients with overt primary hypothyroidism was higher than the other studies done in similar settings. Community-based studies of the prevalence of diabetes in patients with hypothyroidism can be done.
